# Gga-miR-19b-3p Inhibits Newcastle Disease Virus Replication by Suppressing Inflammatory Response via Targeting RNF11 and ZMYND11

**DOI:** 10.3389/fmicb.2019.02006

**Published:** 2019-08-27

**Authors:** Yu Chen, Wen Liu, Haixu Xu, Jingjing Liu, Yonghuan Deng, Hao Cheng, Tiansong Zhan, Xiaolong Lu, Tianxing Liao, Lili Guo, Shanshan Zhu, Yuru Pei, Jiao Hu, Zenglei Hu, Xiaowen Liu, Xiaoquan Wang, Min Gu, Shunlin Hu, Xiufan Liu

**Affiliations:** ^1^Animal Infectious Disease Laboratory, College of Veterinary Medicine, Yangzhou University, Yangzhou, China; ^2^Jiangsu Co-innovation Center for Prevention and Control of Important Animal Infections Diseases and Zoonoses, Yangzhou University, Yangzhou, China; ^3^Jiangsu Key Laboratory of Zoonosis, Yangzhou University, Yangzhou, China

**Keywords:** NDV, gga-miR-19b-3p, DF-1 cells, RNF11, ZMYND11, inflammatory cytokine, NF-κB signaling

## Abstract

Newcastle disease (ND), an acute and highly contagious avian disease caused by virulent Newcastle disease virus (NDV), often results in severe economic losses worldwide every year. Although it is clear that microRNAs (miRNAs) are implicated in modulating innate immune response to invading microbial pathogens, their role in host defense against NDV infection remains largely unknown. Our prior study indicates that gga-miR-19b-3p is up-regulated in NDV-infected DF-1 cells (a chicken embryo fibroblast cell line) and functions to suppress NDV replication. Here we report that overexpression of gga-miR-19b-3p promoted the production of NDV-induced inflammatory cytokines and suppressed NDV replication, whereas inhibition of endogenous gga-miR-19b-3p expression had an opposite effect. Dual-luciferase and gene expression array analyses revealed that gga-miR-19b-3p directly targets the mRNAs of ring finger protein 11 (RNF11) and zinc-finger protein, MYND-type containing 11 (ZMYND11), two negative regulators of nuclear factor kappa B (NF-κB) signaling, in DF-1 cells. RNF11 and ZMYND11 silencing by small interfering RNA (siRNA) induced NF-κB activity and inflammatory cytokine production, and suppressed NDV replication; whereas ectopic expression of these two proteins exhibited an opposite effect. Our study provides evidence that gga-miR-19b-3p activates NF-κB signaling by targeting RNF11 and ZMYND11, and that enhanced inflammatory cytokine production is likely responsible for the suppression of NDV replication.

## Introduction

Newcastle disease virus (NDV) was first described in the early 1900s as the contagious agent of the fatal avian disease known as chicken pest (Dimitrov et al., 2016, 2017). It is classified as an avian paramyxovirus-1 (APMV-1) in the Avulavirus genus of the family Paramyxoviridae (Cheng et al., 2016). The viral particles have a negative-sense, single-stranded RNA genome of about 15 kb that contains six genes in the order of 3′-NP-P-M-F-HN-L-5′, encoding six proteins: nucleoprotein (NP), phosphor protein (P), matrix protein (M), fusion protein (F), hemagglutinin-neuraminidase (HN), and large protein (L), respectively. As one of the most devastating pathogens for poultry industry, NDV has a wide host range and can naturally or experimentally infect more than 250 bird species (Ganar et al., 2014). Although all NDV isolates belong to a single serotype, they have a high genetic diversity and evolve rapidly. According to recent epidemiological investigations, sub-genotype VIId has become the dominant genotype in many Asian and African countries since the late 1990s (Qin et al., 2008; Wu et al., 2011; Munir et al., 2012). Currently, vaccination is the main way to prevent and control NDV prevalence. However, due to the wide range of host and high genetic diversity, the failure of Newcastle disease (ND) vaccine immunization and reduction of vaccine efficiency often occur in cultivation industry worldwide (Miller et al., 2009, 2013).

During NDV infection, a variety of the host pattern recognition receptors (PRRs) are activated such as toll-like receptor 3 (TLR-3) (Cheng et al., 2014), retinoic-acidinducible gene I (RIG-I) (Kato et al., 2006; Sun et al., 2013), melanoma differentiation associated gene 5 (MDA5) (Rue et al., 2011), and protein kinase R (PKR) (Liao et al., 2016) by recognizing virus double-stranded RNA. These PRRs subsequently activate downstream transcription factors such as interferon regulatory factor 7 (IRF7) (Wang et al., 2014) and NF-κB (Rajmani et al., 2016), leading to inflammatory cytokine production. For NF-κB activation, PRRs initiate a signaling cascade resulting in the IκB kinase (IKK) phosphorylation, which subsequently phosphorylates and ubiquitinates IκB-α bound to NF-κB in resting cells, and results in IκB-α proteasomal degradation, thereby allowing NF-κB nuclear translocation to transcriptionally activate its gene targets (Woronicz et al., 1997). However, to maintain cellular homeostasis, activation of NF-κB signaling pathway is tightly controlled with multiple tiers of regulation (Ruland, 2011), such as RNF11 (Shembade et al., 2009; Dalal et al., 2012; Pranski et al., 2012) and ZMYND11 (Ikeda et al., 2009, 2010). These two proteins negatively regulate NK-κB activity and greatly impact the replication of certain viruses such as Hendra virus (HeV) (Stewart et al., 2013), Japanese encephalitis virus (JEV) (Ashraf et al., 2016), and Epstein-Barr virus (EBV) (Ikeda et al., 2009).

miRNAs are small non-coding RNAs that are usually composed of 21–23 nucleotides. miRNAs negatively regulate the expression of half protein-coding genes involving in cell growth, proliferation, apoptosis, and signal transduction through post-transcriptional mechanisms (Bartel, 2004). Accumulating evidence suggests that virus infection profoundly impacts miRNA expression, and that altered miRNA expression can enhance or suppress anti-viral responses (Trobaugh and Klimstra, 2017). Recent studies have shown that NDV infection alters the levels of several miRNAs. For example, NDV infection induces hsa-miR-485 expression in human embryonic kidney (HEK) 293T cells, which in turn suppresses antiviral response and enhances NDV replication by targeting RIG-I mRNA (Ingle et al., 2015). In contrast, NDV infection down-regulates gga-miR-203a expression in chicken embryos, overexpression of gga-miR-203a effectively increases NDV replication both *in vivo* and *in vitro* via targeting transglutaminase 2 (TGM2) (Jia et al., 2018). Up-regulated gga-miR-375 in NDV-infected DF-1 cells suppresses NDV replication via targeting viral M gene or cellular embryonic lethal, abnormal vision, Drosophila-like RNA binding protein 4 (ELAVL4) gene (Wang et al., 2019). These observations collectively suggest that miRNAs play a critical role in NDV infection and replication. Identification of host-dysregulated miRNAs and understanding of their roles in virus replication may shed light on NDV pathogenesis and provide better strategies for the control of NDV infection.

Our present study focuses on the effect of gga-miR-19b-3p on NDV replication and the underlying molecular mechanisms. Here we report that RNF11 and ZMYND11 are two targets of gga-miR-19b-3p in DF-1 cells, and that up-regulation of gga-miR-19b-3p and the subsequent down-regulation of RNF11 and ZMYND11 expression enhance NDV-induced NF-κB activation, leading to increased inflammatory cytokine production and suppression of virus replication. Data presented herein improve our understanding of the role of host miRNAs in regulating NDV replication.

## Materials and Methods

### Cells and Viruses

DF-1 cells obtained from ATCC (CRL-12203) were cultured in Dulbecco’s modified Eagle’s medium (DMEM) (Life Technologies, United States) supplemented with 10% fetal bovine serum (FBS) (Life Technologies, United States), 100 U/mL penicillin and 100 μg/mL streptomycin at 37°C under 5% CO_2_ atmosphere. Velogenic genotype VII NDV strain JS5/05 (Accession Number: JN631747) was propagated in chicken embryos and the biological characteristics of the virus were determined previously (Hu et al., 2011). Velogenic genotype IV Herts/33 (Accession Number: AY741404) was obtained from Dr. D. J. Alexander (Animal Health and Veterinary Laboratories Agency, United Kingdom) and avirulent genotype II NDV strain La Sota (Accession Number: AF077761) was isolated and maintained in our laboratory.

### MiRNA Mimic and Inhibitor

Gga-miR-19b-3p mimic was chemically modified double-stranded oligonucleotides for overexpression of gga-miR-19b-3p and gga-miR-19b-3p inhibitor was single-stranded oligonucleotides for inhibition of gga-miR-19b-3p. All RNA oligonucleotides were designed and synthesized by GenePharma, China. For miRNA transfection, DF-1 cells were transfected with gga-miR-19b-3p mimic and inhibitor and mimic negative control (mimic-NC) and inhibitor negative control (inh-NC) at a final concentration of 100 nM using EL Transfection Reagent (Transgen Biotech, China) following the manufacturer’s instructions.

### Knockdown of RNF11 and ZMYND11

siRNAs (double-stranded RNA oligonucleotides) (Genepharma, China) were used to knockdown the expressions of RNF11 and ZMYND11 in DF-1 cells and the sequences were shown in Table 1. siRNA duplexes were transfected into DF-1 cells at a final concentration of 50 nM using EL Transfection Reagent (Transgen Biotech, China). The interference efficiency was detected by qRT-PCR and Western blot as described below.

**TABLE 1:** siRNA sequences.

**siRNAs**	**Sense (5′-3′)**	**Anti-sense (5′-3′)**
SiRNA-ZMYND 11-1	GCACAUACCUGCGGUUCAUTT	AUGAACCGCAGGUAUGUGCTT
SiRNA-ZMYND 11-2	GCUAUCCUUGUAUACCUAATT	UUAGGUAUACAAGGAUAGCTT
SiRNA-ZMYND 11-3	GCUGUUGAUGUUCCUACUATT	UAGUAGGAACAUCAACAGCTT
SiRNA-RNF11-1	GCCAGAAAUUAGGAAUUAATT	UUAAUUCCUAAUUUCUGGCTT
SiRNA-RNF11-2	CCUACCGAAGGGAGUCUAUTT	AUAGACUCCCUUCGGUAGGTT
SiRNA-RNF11-3	CCACCUGGACUGUAUAGAUTT	AUCUAUACAGUCCAGGUGGTT
SiRNA-NC	UUCUCCGAACGUGUCACGUTT	ACGUGACACGUUCGGAGAATT

### Determination of Viral Titers

DF-1 cells (2 × 10^5^ cells/mL) were seeded on 12-well plates and cultured for 24 h before transfection with indicated RNA oligonucleotides or plasmids. Eighteen hours after transfection, DF-1 cells were infected with JS5/05 strain at a multiplicity of infection (MOI) of 0.1. The culture supernatants were collected and replaced with an equal volume of fresh media at different time points (12, 24, 36, 48, and 60 h) after infection. The viral contents in the supernatants were quantified by 50% tissue culture infective doses (TCID_50_) on DF-1 cells (Reed and Muench, 1938).

### Plasmids Construction

DNA sequences of gga-miR-19b-3p promoter were cloned from genomic DNA of DF-1 cells using specific primers and sub-cloned into *Kpn*I*/Xho*I restriction sites in the pGL-6 reporter vector (Beyotime, China) to generate the pGL-6-miR-19b-3p reporter vector. Target sequences of gga-miR-19b-3p in RNF11 and ZMYND11 were amplified by polymerase chain reaction (PCR) using a cDNA template synthesized from total RNA of DF-1 cells. Subsequently, both PCR products were sub-cloned into *Xho*I*/Not*I restriction sites in the pmiR-RB-reporter^TM^ vector (Ribobio, China), which can express firefly luciferase and renilla luciferase in one plasmid, to generate pmiR-RNF11-3′UTR-wild and pmiR-ZMYND11-3′UTR-wild plasmids. To generate gga-miR-19b-3p target-mutated reporter vector, pmiR-RNF11-3′UTR-mut and pmiR-ZMYND11-3′UTR-mut were achieved by changing the gga-miR-19b-3p binding seed sequences using Fast Mutagenesis System kit (Transgen Biotech, China) according to manufacturer’s instructions. The coding sequences of RNF11 (Accession No. NM001006540) and ZMYND11 (Accession No. XM025148320) were amplified and subsequently cloned into pCMV-blank overexpression vector (Beyotime, China) to generate pCMV-RNF11 and pCMV-ZMYND11 plasmids. All the primers for plasmids construction (shown in Table 2) were synthesized by Sangon Company, China. All the fragments were amplified by PCR using TransStart Fast Pfu Fly DNA Polymerase (Transgen Biotech, China).

**TABLE 2:** Primers used for plasmids construction.

**Gene**	**Forward primers (5′-3′)**	**Reverse primer (5′-3′)**
19b-3p promoter	GGTACCGCCGGGAGG GTCTGCC	CTCGAGTTCAATTTAAC AACAAATTGCAAA
RNF11-3′UTR-wild	CTCGAGGCCAGGGTTTCTC CGTGAAC	GCGGCCGCCAAGAGATG CGGTATCCTTTGC
ZMYND11-3′UTR-wild	CTCGAGGAGCCATTCCTCCG CTAGAACA	GCGGCCGCCTCGGTCTA AGACCAGCCTCTG
RNF11-3′UTR-mut	ATAGTTTTAAGCTTGTGCAC GTAGTTCTTTT	ACGTGCACAAGCTTAAAAC TATTTCTTTGT
ZMYND11-3′UTR-mut	TTCCCATTAAGGTGCAGCCT TTAAACA	GCACCTTAATGGGAAGC AAATTCTAAACA
RNF11 CDS	ACTAGTATGGGGAACTGCCTC AAGTCC	GGGCCCTCAGTTAGTCTCG TATGATGAAAG
ZMYND11 CDS	ACTAGTATGGCACGCTTA ACGAAGAGACG	GGGCCCTCATCTTTTTCTG CGGCAGGTACG

### miRNA Target Prediction and Dual-Luciferase Reporter Gene Assays

Gga-miR-19b-3p targets in host cells were predicted by two online softwares: TargetScan^1^ and miRDB^2^. GO Ontology (GO) analysis was conducted to screen the genes on the Database for Annotation, Visualization and Integrated Discovery (DAVID) platform. Luciferase activity was measured using Dual-GLO^®^ Luciferase Assay System Kits (Promega, United States) following the manufacturer’s instructions. For targets verification, DF-1 cells were seeded on 24-well plates with a density of 1 × 10^5^ cells/mL per well. After 24 h, the cells were co-transfected with indicated plasmids and RNA oligonucleotides using EL Transfection Reagent. Forty-eight hours after transfection, luciferase assays were performed with a Fluorescence/Multi-Detection Microplate Reader (Biotek, United States). Renilla luciferase activities were normalized on the basis of firefly luciferase activities. For the detection of promoter activity, DF-1 cells were transfected with pGL-6 or pGL-6-miR-19b-3p plasmid using EL Transfection Reagent. To normalize transfection efficiency, pRL-TK renilla luciferase reporter plasmid (Promega, United States) was also transfected into the cells as an internal control. Then the cells were infected with JS 5/05 at different MOIs at 48h post transfection. Twelve hours post infection (hpi), the dual-luciferase activity was detected and firefly luciferase activities were normalized to renilla luciferase activities. To measure the activity of NF-κB, pNF-κB-luc (Beyotime, China) plasmid and indicated siRNAs or plasmids were transfected into DF-1 cells together with the internal control plasmid pRL-TK (Promega, United States). Eighteen hours after transfection, the cells were infected with JS 5/05 strain at an MOI of 0.1 and dual-luciferase assays were performed as described above at 18 hpi.

### miRNA and Total RNA Extraction and Quantitative Real-Time PCR (qRT-PCR) Analysis

To measure the dynamic expression pattern of gga-miR-19b-3p after NDV infection, DF-1 cells seeded in 12-well plates were infected with JS5/05 strain at different MOIs for 12 h and then miRNAs were extracted using a miRNA Extraction Kit according to manufacturer’s instructions. cDNA synthesis was carried out with a tagged polyT primer according to the protocol of One Step miRNA cDNA Synthesis Kit. Quantification of gga-miR-19b-3p was performed by HG miRNA SYBR Green PCR Kit with a pair of specific primers. Gga-miR-19b-3p expression level was normalized to 5s rRNA levels using the 2^–Δ^ ^Δ^ ^*Ct*^ model. All kits used for miRNA extraction, cDNA synthesis and qRT-PCR were purchased from HaiGene Corporation, China.

For the detection of inflammatory cytokines and IκB-α, DF-1 cells were transfected with indicated RNA oligonucleotides for 18 h before infected with JS 5/05 at an MOI of 0.1. Then the total RNAs were extracted from the cells at 18 hpi using TRIzol (TransGen Biotech, China) following the manufacturer’s instructions. The cDNA synthesis and qRT-PCR reaction were performed using TransScript Green One-Step qRT-PCR Super Mix (TransGen Biotech, China) according to the manufacturer’s instructions. To detect RNF11 and ZMYND11 mRNA expression levels, DF-1 cells were transfected with indicated RNA oligonucleotides or infected with JS 5/05 or Herts/33 or La Sota strain at an MOI of 0.1. Eighteen hours post transfection or infection, qRT-PCR were performed as described above using RNF11 and ZMYND11 specific primers. The relative expressions of inflammatory cytokines, IκB-α, RNF11, and ZMYND11 were all normalized with glyceraldehyde phosphate dehydrogenase (GAPDH) and calculated using the 2^–ΔΔ*CT*^ method. All qRT-PCR experiments were preformed in LightCycler 480 (Roche, Switzerland) and primers used were shown in Table 3.

**TABLE 3:** Primers used for qRT-PCR.

**Gene**	**Forward primers (5′-3′)**	**Reverse primer (5′-3′)**
gga-miR-19b-3p	AGTGTGCAAATCCATGCAA	GGTCCAGTTTTTTTTTTTTTTTCAGT
IFN-β	TGCACAGCATCCTACTG CTCTTG	GTTGGCATCCTGGTGACGAA
TNF-α	GGACAGCCTATGCCAACAAG	ACACGACAGCCAAGTCAACG
IL-1β	ACCCGCTTCATCTTCTACCG	TCAGCGCCCACTTAGCTT
IL-6	GGCATTCTCATTTCCTTCT	CTGGCTGCTGGACATTTT
IL-8	CCAAGCACACCTCTCTTCCA	GCAAGGTAGGACGCTGGTAA
IκB-α	CTGGACTCCATGAAGGA GGAGG	TCGTGAATGATCGCCA AGTGGAG
RNF11	GCCTTGACCCACCATTGTCTTT	CCTTGACCCAAATGCCAGGAC
ZMYND11	GGCGATCCAACATCTTTGGGC	GCCTTTGACCCTTTACACCCCA
5s RNA	AACGCCCGATCTCGTCTGAT	AGTCTCCCATCCAAGTA CTAACCG
GAPDH	GAGGGTAGTGAAGGCTGCTG	CACAACACGGTTGCTGTATC

### Enzyme-Linked Immunosorbent Assay (ELISA)

DF-1 cells in 6-well plates were transfected with indicated RNA oligonucleotides or plasmids and were incubated for 18 h and then either left uninfected or infected with JS 5/05 at an MOI of 0.1 for 18 h. The protein levels of IFN-β, TNF-α, IL-1β, IL-6, and IL-8 in cell cultures were determined by ELISA kits according to the manufacturer’s instructions. The IFN-β (cat. no. CH50022), TNF-α (cat. no. CH50032), IL-1β (cat. no. CH50020), IL-6 (cat. no. CH50016), and IL-8 ELISA kits (cat. no. CH50027) were purchased from Bio-Swamp, China.

For the detection of phosphorylation of IκB-α protein, DF-1 cells seeded on 12-well plates were transfection with indicated RNA oligonucleotides or plasmids for 18 h. Then the cells were infected with JS5/05 strain at an MOI of 0.1 for 18 h. The phosphorylation of IκB-α protein was measured by ELISA kit (SbjBio, China, cat. no. SBJ-C134) according to the manufacturer’s instructions.

### Western Blot Analysis

To measure the expression levels of RNF11 and ZMYND11 proteins, DF-1 cells were transfected with indicated RNA oligonucleotides or plasmids or infected with different NDV strains at an MOI of 0.1. Eighteen hours post transfection, the cells were lysed and 20 μg protein samples of each group were used for electrophoresis on 12% sodium dodecylsulfate-polyacrylamide gel electrophoresis (SDS-PAGE) gels, and resolved proteins were transferred onto polyvinylidene difluoride (PVDF) membranes using electro-transfer (Bio-Rad, United States). After blocking with 5% skimmed milk for 1 h at room temperature, the membranes were incubated with the appropriate primary antibodies, including rabbit polyclonal anti-RNF11 (LSBio, United States), rabbit polyclonal anti-ZMYND11 (LSBio, United States) and mouse monoclonal anti-β-actin (Transgene, China) followed by incubation with goat anti-rabbit and goat anti-mouse HRP-conjugated secondary antibodies (Beyotime, China). The proteins on the membranes were detected using an ECL^TM^ detection system (Bio-Rad, United States).

To measure the translocation of NF-κB from the cytoplasm to the nucleus, DF-1 cells transfected with indicated RNA oligonucleotides or plasmids were infected with JS 5/05 at an MOI of 0.1 for 18 h. Then cytoplasmic and nuclear proteins of the cells were prepared using nuclear and cytoplasmic protein extraction kit (Beyotime, China) according to the manufacturer’s instructions. Western blot assays were performed as described above using NF-kB p65 rabbit monoclonal antibody (Beyotime, China). Monoclonal rabbit anti-tubulin β and anti-histone H3 antibody (Beyotime, China) were used as control of cytoplasmic and nuclear proteins, respectively. The relative protein quantification was analyzed by Image J software (Bethesda, United States).

### Statistical Analysis

All data are the averages of triplicates and are representative of results from at least three independent experiments. The statistical analysis were analyzed using one-way analysis of variance (ANOVA) following Bonferroni’s multiple comparison tests for TCID_50_, miRNA, Western blot and mRNA detections and two-way ANOVA using a Bonferroni’s multiple comparison tests for viral growth curve using GraphPad Prism 5 (GraphPad Software, San Diego, United States). *P* < 0.05 was considered as a significant difference.

## Results

### Gga-miR-19b-3p Is Upregulated During NDV Infection and Inhibits NDV Replication in DF-1 Cells

**FIGURE 1:**
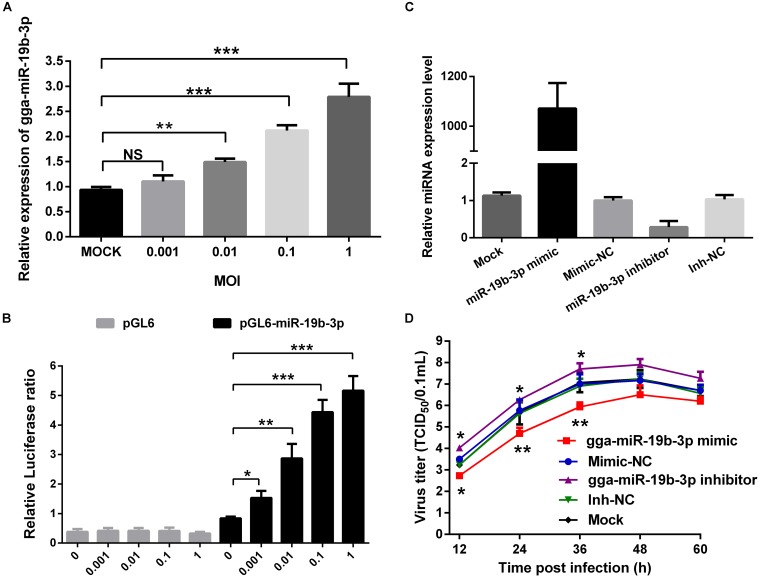
Newcastle disease virus-induced gga-miR-19b-3p inhibits NDV replication in DF-1 cells. **(A)** DF-1 cells were infected with JS 5/05 strain at indicated MOIs for 12 h, and then the expression of gga-miR-19b-3p was detected by qRT-PCR. **(B)** The pGL-6-miR-19b-3p or pGL-6 plasmid was transfected into DF-1 cells together with pRL-TK renilla luciferase reporter plasmid as an internal control. Forty-eight hours post transfection, the cells were infected with JS 5/05 strain at different MOIs for 12 h, and then the dual-luciferase activity was detected and firefly luciferase activities were normalized to firefly renilla luciferase activities. **(C,D)** DF-1 cells were transfected with gga-miR-19b-3p mimic or inhibitor or mimic-NC or inh-NC at a final concentration of 100 nM or left untreated. The gga-miR-19b-3p expression was measured by qRT-PCR at 18 h post transfection **(C)**. Eighteen hours after transfection, DF-1 cells were infected with JS5/05 strain at an MOI of 0.1. The viral road in the supernatants collected at different time points were quantified by TCID_50_ on DF-1 cells **(D)**. Results are representative of three independent experiments and presented as means ± SD. ^∗^*p* < 0.05, ^∗∗^*p* < 0.01, ^∗∗∗^*p* < 0.001.

We first conducted qRT-PCR to determine if NDV infection regulated gga-miR-19b-3p expression in DF-1 cells. As shown in Figure 1A, gga-miR-19b-3p was up-regulated in NDV-infected DF-1 cells in a dose-dependent manner. Consistently, NDV infection also increased the gga-miR-19b-3p promoter-driven luciferase reporter gene expression in a dose-dependent manner (Figure 1B).

We next evaluated the effect of gga-miR-19b-3p on NDV replication. DF-1 cells were transfected with gga-miR-19b-3p mimic or its inhibitor, followed by infection with NDV (JS 5/05 strain, 0.1 MOI). As shown in Figure 1C, gga-miR-19b-3p mimic increased gga-miR-19b-3p expression about 1000-fold in DF-1 cells, whereas gga-miR-19b-3p antagonist decreased its expression by >70%. Overexpression of gga-miR-19b-3p mimic significantly suppressed NDV replication, whereas gga-miR-19b-3p inhibitor increased NDV replication (Figure 1D).

### Gga-miR-19b-3p Positively Regulates NDV-Induced Expression of Inflammatory Cytokines

**FIGURE 2:**
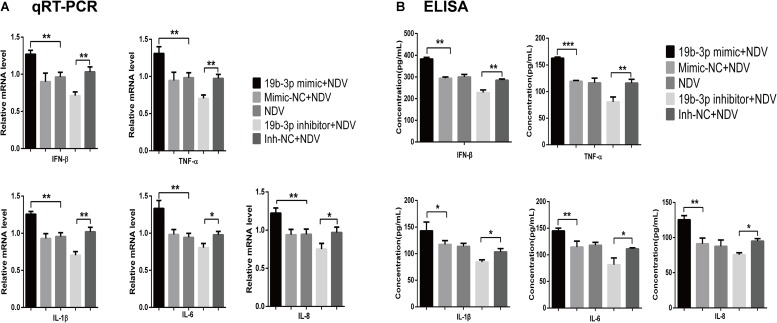
Gga-miR-19b-3p promotes NDV-induced expression of inflammatory cytokines. DF-1 cells were transfected with indicated RNA oligonucleotides at a final concentration of 100 nM for 18 h or left untreated before infected with JS 5/05 at an MOI of 0.1. The expression levels of IFN-β, TNF-α, IL-1β, IL-6, and IL-8 were measured by qRT-PCR **(A)** and ELISA **(B)** at 18 hpi. Results are representative of three independent experiments and presented as means ± SD. ^∗^*p* < 0.05, ^∗∗^*p* < 0.01, ^∗∗∗^*p* < 0.001.

We next determined if gga-miR-19b-3p affected virus replication by regulating the inflammatory cytokine production. As shown in Figures 2A,B, gga-miR-19b-3p mimic significantly increased both mRNA and protein levels of IFN-β, TNF-α, IL-1β, IL-6, and IL-8 in NDV-infected DF-1 cells. In contrast, gga-miR-19b-3p inhibitor significantly decreased the levels of IFN-β, TNF-α, IL-1β, IL-6, and IL-8 mRNA and protein.

### Gga-miR-19b-3p Directly Targets RNF11 and ZMYND11 in DF-1 Cells

We then investigated the targets of gga-miR-19b-3p that might be involved in regulating NDV-induced inflammatory cytokine production. A total of 571 and 547 targets were predicted by TargetScan and miRDB, respectively. Two hundred and twenty-one common target genes were used to do GO analysis on the DAVID platform. After enrichment by GO analysis, we found that two potential target genes RNF11 and ZMYND11were clustered on immune system process. Moreover, both of them were reported as negatively regulators of NK-κB activity. Finally, RNF11 and ZMYND11 were screened for validation given the GO analysis results and previous study.

**FIGURE 3:**
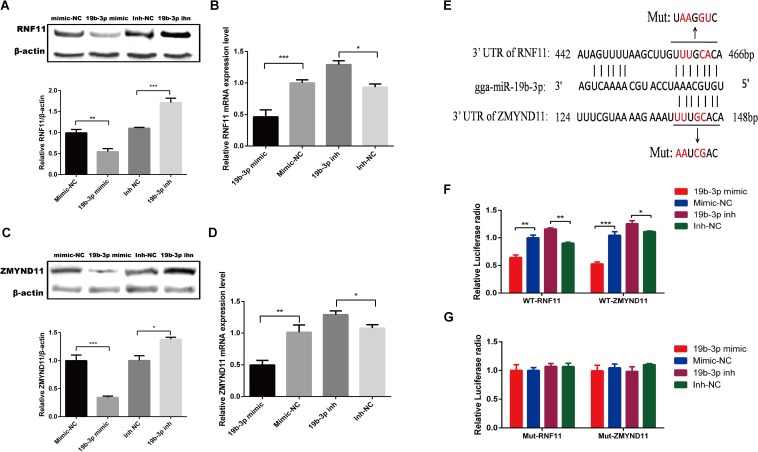
Gga-miR-19b-3p directly targets RNF11 and ZMYND11 in DF-1 cells. DF-1 cells were transfected with indicated RNA oligonucleotides at a final concentration of 100 nM for 18 h. Then the expression of RNF11 and ZMYND11 were detected by Western blot **(A,C)** and qRT-PCR **(B,D)** assays. The relative levels of RNF11 and ZMYND11 proteins were calculated as follows: band density of RNF11 or ZMYND11/band density of β-actin in the same sample and showed in the below of **(A,C)**. Diagram of the predicted target sites for gga-miR-19b-3p in RNF11 and ZMYND11 were shown in **(E)**. DF-1 cells were co-transfected with indicated RNA oligonucleotides and wild type **(F)** or mutant luciferase reporter gene vectors **(G)** before luciferase reporter gene assay was performed. The relative level of renilla luciferase activities was calculated normalized on the basis of firefly luciferase activities. Data are representative of three independent experiments and presented as means ± SD. ^∗^*p* < 0.05, ^∗∗^*p* < 0.01, ^∗∗∗^*p* < 0.001.

To examine whether gga-miR-19b-3p directly targets RNF11 and ZMYND11, We first detected whether gga-miR-19b-3p regulates the RNF11 and ZMYND11 expression. qRT-PCR and Western blot revealed that transfection of DF-1 cells with gga-miR-19b-3p mimic led to decreased RNF11 (Figures 3A,B) and ZMYND11 (Figures 3C,D) expression at both protein and mRNA levels, compared to that transfected with mimic-NC or inh-NC. We then constructed luciferase reporter plasmids containing the wild-type or mutant 3′-UTR of the RNF11 and ZMYND11 genes. Predicted target sequences for gga-miR-19b-3p in the 3′-UTR of RNF11 and ZMYND11 are illustrated in Figure 3E. DF-1 cells were co-transfected with indicated RNA oligonucleotides plus wild-type or mutated-type luciferase reporter plasmids. Forty eight hours after transfection, dual-luciferase reporter assays were performed. gga-miR-19b-3p mimic significantly decreased the ratio of Renilla/Firefly in pmiR-RNF11-3′UTR-wild and pmiR-ZMYND11-3′UTR-wild transfected DF-1 cells, but not in pmiR-RNF11-3′UTR-mut and pmiR-ZMYND11-3′UTR-mut cells (Figures 3F,G). As expected, inhibition of gga-miR-19b-3p exhibited a significant promotion of the luciferase radio in pmiR-RNF11-3′UTR-wild and pmiR-ZMYND11-3′UTR-wild DF-1 cells, whereas no changes in pmiR-RNF11-3′UTR-mut and pmiR-ZMYND11-3′UTR-mut cells (Figures 3F,G). Combined with these results, we demonstrated that RNF11 and ZMYND11 are directly targeted by gga-miR-19b-3p in DF-1 cells.

### NDV Infection Inhibits the Expressions of RNF11 and ZMYND11

**FIGURE 4:**
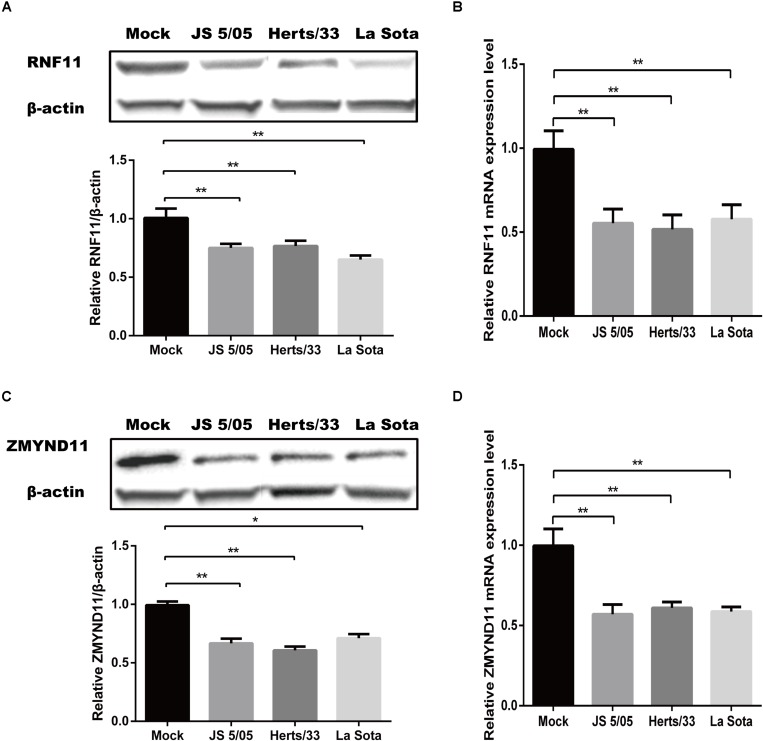
Newcastle disease virus infection inhibits the expressions of RNF11 and ZMYND11. DF-1 cells were infected with different NDV strains (JS 5/05, Herts/33, and La Sota) at an MOI of 0.1 for 18 h. The expressions of RNF11 and ZMYND11 were detected by Western blot **(A,C)** and qRT-PCR **(B,D)** assays. The relative levels of RNF11 and ZMYND11 proteins were calculated as described above and showed in the below of **(A,C)**. Data are representative of three independent experiments and presented as means ± SD. ^∗^*p* < 0.05, ^∗∗^*p* < 0.01.

Given that gga-miR-19b-3p directly targeted RNF11 and ZMYND11 mRNAs, we tested if NDV infection indeed affected the expression of these two proteins. qRT-PCR and Western blot revealed that the protein and mRNA levels of RNF11 (Figures 4A,B) and ZMYND11 (Figures 4C,D) were significantly down-regulated in DF-1 cells infected with three different NDV strains, including JS 5/05, Herts/33 and La Sota.

### RNF11 and ZMYND11 Modulate NDV Replication via Suppressing NDV-Induced Inflammatory Cytokines

**FIGURE 5:**
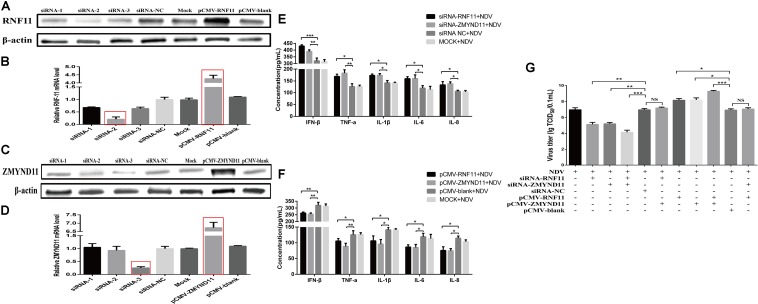
RNF11 and ZMYND11 inhibit NDV-induced inflammatory cytokines and promote NDV replication in DF-1 cells. DF-1 cells were transfected with indicated siRNAs or siRNA-NC or overexpression plasmids or pCMV-blank. The efficiencies of interference and overexpression in transfected DF-1 cells were measured by Western blot **(A,C)** and qRT-PCR **(B,D)** at 18 h post transfection. DF-1 cells transfected with indicated siRNAs or siRNA-NC or overexpression plasmids or pCMV-blank or left untreated were infected with JS 5/05 at an MOI of 0.1 for 18 h. The expression levels of IFN-β, TNF-α, IL-1β, IL-6, and IL-8 were measured by ELISA assay **(E,F)**. DF-1 cells transfected with indicated siRNAs or siRNA-NC or overexpression plasmids or pCMV-blank were infected with JS 5/05 at an MOI of 0.1. After 36 h, viral titers in infected DF-1 cells were measured by TCID_50_ as described above **(G)**. Data are representative of three independent experiments and presented as means ± SD. ^∗^*p* < 0.05, ^∗∗^*p* < 0.01, ^∗∗∗^*p* < 0.001.

We next determined if RNF11 and ZMYND11 indeed regulated inflammatory production and virus replication. Firstly, we detected the interference and overexpression efficiency of siRNAs and overexpression plasmids by Western blot and qRT-PCR assays. As shown in Figures 5A–D, the levels of RNF11 or ZMYND11 were increased by 4.5 or 7-fold in DF-1 cells transfected with pCMV-RNF11 and pCMV-ZMYND11, respectively. siRNA-RNF11-2 (hereafter called siRNA-RNF11) and siRNA-ZMYND11-3 (hereafter called siRNA-ZMYND11) down-regulated the expression of ZMYND and RNF11 by about 70%. RNF11 or ZMYND11 knockdown in DF-1 cells markedly enhanced the expressions of NDV-induced inflammatory cytokines and suppressed NDV replication, whereas RNF11 or ZMYND11 overexpression blocked NDV-induced inflammatory cytokine expression and promoted NDV replication (Figures 5E–G). Simultaneous knockdown or overexpression of these two proteins showed synergistic effects on viral replication. However, overexpression of ZMYND11 and knockdown of RNF-11, or overexpression of RNF-11 and knockdown of ZMYND11 showed no effect on viral replication.

### RNF11 and ZMYND11 Suppress NF-κB Activity

**FIGURE 6:**
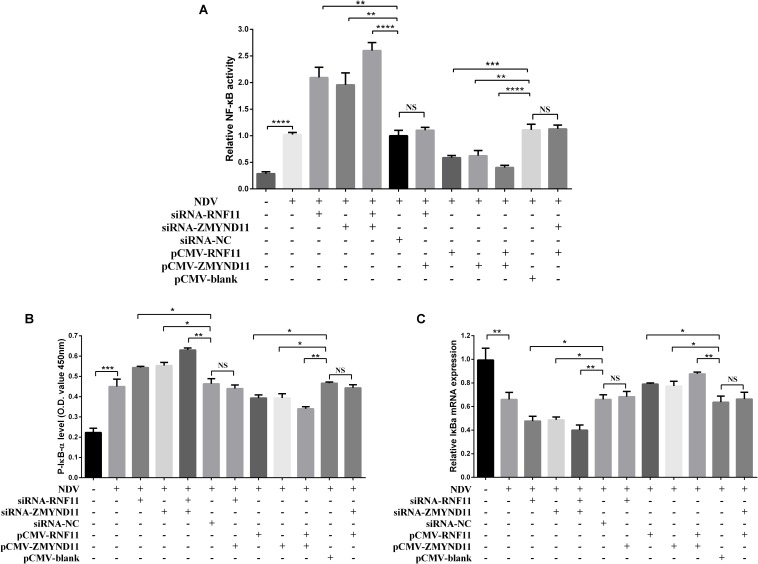
RNF11 and ZMYND11 suppress NF-κB activity. pNF-κB-luc plasmid and indicated siRNAs or siRNA-NC or overexpression plasmids or pCMV-blank were co-transfected into DF-1 cells together with the internal control plasmid pRL-TK. Eighteen hours after transfection, the cells were infected with JS 5/05 strain at an MOI of 0.1 or left uninfected, dual-luciferase **(A)**, ELISA **(B)**, and qRT-PCR **(C)** assays were performed as described above at 18 hpi. NF-κB activities were indicated by the ratio of firefly luciferase activities to renilla luciferase activities. The relative expression of IκB-α was normalized with GAPDH and calculated using the 2^–Δ^ ^Δ^ ^CT^ method. Data are representative of three independent experiments and presented as means ± SD. ^∗^*p* < 0.05, ^∗∗^*p* < 0.01, ^∗∗∗^*p* < 0.001, ^*⁣*⁣**^p < 0.0001.

**FIGURE 7:**
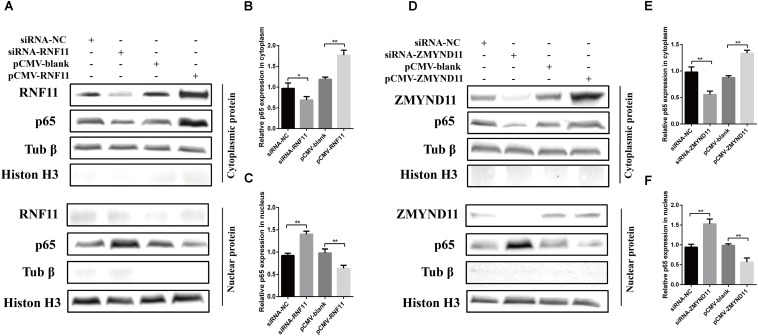
RNF11 and ZMYND11 inhibit the nucleus translocation of p65. DF-1 cells were transfected with indicated siRNAs or siRNA-NC or overexpression plasmids or pCMV-blank for 18 h. Then JS 5/05 strain were used to infect the cells at an MOI of 0.1. Eighteen hours post infection, the cytoplasmic and nuclear proteins of the cells were prepared as described above. Western blot was used to detect the translocation of p65 from cytoplasm to the nucleus **(A,D)**. The relative p65 expression in cytoplasm **(B,E)** or nucleus **(C,F)** was analyzed by Image J software and calculated normalized on the basis of tubulin β or histone H3, respectively. Data are representative of three independent experiments and presented as means ± SD. ^∗^*p* < 0.05, ^∗∗^*p* < 0.01.

Previous studies demonstrated that RNF11 and ZMYND11 negatively regulate the NF-κB signaling pathway in human cell lines (Ikeda et al., 2010; Dalal et al., 2012). In order to explore the mechanism by which RNF11 and ZMYND11 suppress NDV-induced inflammatory cytokines, we detected NF-κB activity in siRNAs or overexpression plasmids transfected DF-1 cells after NDV infection using a pNF-κB-luc plasmid which containing multiple binding motifs of NF-κB (GGGAATTTCC). We found that knockdown of RNF11 or ZMYND11 in DF-1 cells significantly increased NF-κB activity, while overexpression of them had completely opposite effects (Figure 6A). For further understand the underlying mechanism of the suppression effect on NF-κB activity by RNF11 and ZMYND11, we then detected the changes of p-IκB-α, IκB-α, and p65 nuclear translocation in siRNAs or overexpression plasmids transfected DF-1 cells after NDV infection. As shown in Figures 6B,C, knockdown of RNF11 or ZMYND11 in DF-1 cells significantly promoted p-IκB-α expression and inhibited IκB-α expression, while overexpression of them had completely opposite effects. Similarly, simultaneous knockdown or overexpression of these two proteins also showed synergistic effects on NF-κB activity, p-IκB-α and IκB-α expression. However, overexpression of ZMYND11 and knockdown of RNF-11, or overexpression of RNF-11 and knockdown of ZMYND11 showed no effect. In expectation, transfection of siRNA-RNF11 or siRNA-ZMYND11 increased the translocation of p65, an active subunit of NF-κB with transcriptional activity (Vermeulen et al., 2002), from the cytoplasm to the nucleus (Figures 7A,D). In contrast, treatment of cells with pCMV-RNF11 or pCMV-ZMYND11 significantly inhibited the nuclear translocation of p65 in NDV-infected DF-1 cells (Figures 7A,D). The relative p65 expression in cytoplasm or nucleus was calculated normalized on the basis of tubulin β or histone H3, respectively (Figures 7B,C,E,F).

## Discussion

miRNAs are post-transcriptional regulators that mediate translational repression and/or mRNA degradation by complementary binding to the 3′ UTRs in target mRNAs. Mounting evidence suggest that cellular miRNAs can coordinate host defense against viral infections by direct binding to virus genome or through virus-mediated changes in the host transcriptome (Trobaugh and Klimstra, 2017). For some paramyxoviruses, such as respiratory syncytial virus (RSV) (Bakre et al., 2012; Thornburg et al., 2012), HeV (Stewart et al., 2013), and Nipah virus (NiV) (Foo et al., 2016), critical roles of cellular miRNAs in host defense against virus replication have already been well characterized. Even so, little is known about miRNAs roles in NDV replication until now.

Our previous deep sequencing results showed that the expression of gga-miR-19b-3p is significantly increased in NDV-infected DF-1 cells. Further study suggests that this up-regulated miRNAs might function as an anti-viral factor and play a role in NDV-infected DF-1 cells. Our present study focuses on the mechanism by which gga-miR-19b-3p inhibits NDV replication. We found that NDV infection increased the expression and promoter activity of gga-miR-19b-3p. miR-19b-3p has been shown to play an important role in regulating the replication of other viruses. For example, up-regulated miR-19b enhances CD8^+^ T cell function via targeting the phosphatase and tensin homolog (PTEN) and inhibits viral production of human immunodeficiency virus (HIV) in T cells (Yin et al., 2018). Our study showed that transfection of gga-miR-19b-3p mimic significantly promoted NDV-induced inflammatory cytokines and inhibited NDV replication in DF-1 cells, suggesting that gga-miR-19b-3p might play an anti-viral role during NDV infection.

Like other paramyxoviruses, NDV infection can be sensed by host innate immune system via PRRs in the cytosol. Activated PRRs lead to activation of the innate immune response and the production of inflammatory cytokines that establishes an antiviral state (Schoggins and Rice, 2011). NF-κB plays a crucial role in the production of inflammatory cytokines (i.e., TNF-α, IL-6), apoptotic factors, and oxidative stress (Deng et al., 2018). A growing body of research suggests that miRNAs can regulate viral replication via influencing NF-κB activity. For example, has-miR-215 directly targets the tripartite motif 22 (TRIM22) to block the NF-κB signaling pathway, and exerts a positively regulatory role on (hepatitis C virus) HCV replication (Tian and He, 2018). Up-regulated miR-221-5p inhibits porcine epidemic diarrhea virus (PEDV) replication by targeting the 3′ UTR of the viral genome and activating the NF-κB-signaling pathway (Zheng et al., 2018). In addition, during HIV infection, the down-regulation of miR-16 results in the activation of the NF-κB signaling pathway, thus inhibiting HIV replication (Li et al., 2010).

RNF11 is an evolutionarily conserved 154 amino acid protein that acts as a negative regulator of NF-κB signaling pathway through its associations with the A20 ubiquitin-editing protein complex (Pranski et al., 2012). Earlier studies have shown that ZMYND11 or BS69, a multidomain cellular protein, can directly interact with tumor necrosis factor receptor associated factor 3 (TRAF3) to negatively regulated Epstein–Barr virus latent membrane protein 1-mediated NF-κB activation and then enhanced IL-6 expression (Ikeda et al., 2010). Moreover, recent studies demonstrated that ZMYND11 could negatively regulate the NF-κB signaling pathway in mycoplasma gallisepticum-infected chicken embryos and DF-1 cells (Hu et al., 2016). In our present study, we found that RNF11 and ZMYND11were directly targets of gga-miR-19b-3p in DF-1 cells. Since gga-miR-19b-3p enhanced NDV-induced inflammatory response, it was imperative to evaluate effect of RNF11 and ZMYND11 on inflammatory cytokines production in NDV-infected DF-1 cells. We found that knockdown of RNF11 and ZMYND11 increased NDV-induced inflammatory cytokines and inhibited NDV replication, similar to the effect of gga-miR-19b-3p. However, overexpression of RNF11 and ZMYND11 showed an opposite effect. These results suggested that gga-miR-19b-3p enhances NDV-induced inflammatory response via targeting RNF11 and ZMYND11. Moreover, different virulence NDV strains infection resulted in a similar suppression of these two proteins expression, indicated that a virulence-independent regulation of RNF11 and ZMYND11 during NDV infection *in vitro*.

Further research of mechanism by which RNF11 and ZMYND11 influence NDV-induced inflammatory response, we found that silencing of these two proteins by siRNAs promotes NDV-induced NF-κB activity indicated that these two proteins negatively regulate NF-κB signaling pathway in avian cells. It has been well established that phosphorylation and subsequent proteasomal degradation of IκB-α and translocation of NF-κB from the cytoplasm to the nucleus are key determinants of NF-κB activation (Woronicz et al., 1997; Zhu et al., 2015). Therefore, we also determined the effect of RNF11 and ZMYND11 on the expression of p-IκB-α and IκB-α and nucleus translocation of NF-κB after NDV infection in DF-1 cells. We found that treatment of DF-1 cells with siRNA-RNF11 and siRNA-ZMYND11 enhanced the expression of p-IκB-α and nucleus translocation of p65 and inhibited IκB-α expression, whereas its overexpression prevented the expression of p-IκB-α and p65 nuclear translocation and increased IκB-α expression. All these results strongly indicate that RNF11 and ZMYND11can negatively regulate NF-κB activity in DF-1 cells after NDV infection and their down-regulation after NDV infection may be responsible for gga-miR-19b-3p stimulative role on NDV-induced inflammatory cytokines expression. However, the molecular mechanism by which RNF11 and ZMYND11 negatively regulate NF-κB activity in DF-1 cells remains to be further investigated.

To further elucidate the association of the regulated roles of RNF11 and ZMYND11, we knocked down or overexpressed of both proteins concurrently or knocked down one of them while overexpressed the other one before NDV infection. We found that simultaneous knockdown or overexpression of both proteins showed synergistic effects on viral replication and NF-κB activity. However, overexpression of ZMYND11 and knockdown of RNF-11, or overexpression of RNF-11 and knockdown of ZMYND11 showed no effect on viral replication and NF-κB activity. However, we believe that these results do not indicate that RNF11 and ZMYND11 have similar abilities in regulating NDV replication and NF-κB activity. Since the interference efficiency of both genes is approximately 30%, while the overexpression efficiency of both genes is approximately five to sevenfold. Moreover, the expression levels of endogenous RNF11 and ZMYND11 are also different in DF-1 cells. Therefore, further precise experiments are needed to confirm which protein plays more critical role on NDV replication.

In summary, our results indicate that NDV-induced gga-miR-19b-3p inhibits the expressions of RNF11 and ZMYND11 by directly binding them, thereby enhancing NF-κB activity and increasing inflammatory cytokines production, which finally leads to the inhibition of NDV replication in DF-1 cells. However, the study of gga-miR-19b-3p *in vivo* is more complicated than that in DF-1 cells, further studies are needed to elucidate the role of gga-miR-19b-3p in host response to NDV infection.

## Data Availability

All datasets generated for this study are included in the manuscript and/or the supplementary files.

## Author Contributions

YC was responsible for experiment design, data analysis, and writing the manuscript. WL, HX, YD, HC, TZ, XLL, TL, LG, SZ, and YP were responsible for performing the experiments. JL, JH, XW, MG, and XWL were responsible for suggestion during the experiments performance. ZH, SH, and XFL were responsible for revising the manuscript. All authors read and approved the final manuscript.

## Conflict of Interest Statement

The authors declare that the research was conducted in the absence of any commercial or financial relationships that could be construed as a potential conflict of interest.

## Footnotes

1 http://www.targetscan.org/

2 http://www.mirdb.org/
